# The prevalence and county-level socioeconomic correlates of visual impairment and glasses ownership among rural students in Western China

**DOI:** 10.3389/fpubh.2023.1069793

**Published:** 2023-01-19

**Authors:** Hongyu Guan, Yunyun Zhang, Zhijie Wang, Xiangzhe Chen, Jing Xue, Yuxiu Ding

**Affiliations:** Center for Experimental Economics in Education, Shaanxi Normal University, Xi'an, China

**Keywords:** visual impairment prevalence, glasses ownership, county-level socioeconomic status, school-age students, public health

## Abstract

**Objectives:**

Vision health has been a major issue in public health care. The vision problems of children in rural China are of particular concern. Effective policies for children's vision health should consider the heterogeneity of individual health levels and regional differences in healthcare services. This study systematically explored the relationship of student visual impairment and glasses ownership with county-level socioeconomic status (namely, county-level per capita GDP, population density and industrial structure) in rural China.

**Methods:**

The study sample included 99,670 students in grades 4–9 from 445 schools in 13 counties in Shaanxi Province. From 2014 to 2020, students took school-based vision screening tests and completed sociodemographic questionnaires. Multivariate regressions were used for empirical analysis.

**Results:**

The prevalence of visual impairment was 30.4% in the total sample, and the rate of visual impairment in girls (34.3%) was higher than that in boys (27.0%). Among visually impaired students, the rate of glasses ownership was only 39.7%, with girls (40.6%) higher than boys (38.8%). The study also found that the visual impairment was significantly correlated with county-level average income, population density and industrial structure, and the glasses ownership was significantly correlated with population density and industrial structure (all *p* < 0.001).

**Conclusions:**

The study found that the county-level socioeconomic status was positively and significantly correlated with visual impairment and glasses ownership, respectively. The vision health management services for students should be jointly carried out by the education, medical and public health departments, and additional investment is needed to increase the accessibility and affordability of vision care services, with special focus on poor regions and disadvantaged groups, so as to narrow the gap in vision health services.

## Introduction

The correlation between people's health and regional socioeconomic development has been concerned by researchers. Empirical evidence has shown that people in poor regions tend to be in poorer health and have less access to healthcare services than those in more affluent regions. The possible reason is that regions with low socioeconomic development have two distinctive features: low per-capita income level and low population density, both of which will dampen the demand and supply of healthcare services, leading to lower accessibility to health services. Furthermore, health inequality between regions can also further exacerbate socioeconomic inequalities between regions.

With regard to regional differences in health outcomes and healthcare-seeking behaviors, refractive errors among children in rural China is of particular concern. Visual impairment is ranked as the third most common disability worldwide (1). According to the Global Burden of Diseases, Injuries, and Risk Factors Study, 223.4 million people worldwide had visual impairment (hereinafter referred to as “VI”) as in 2010, of whom 191 million had moderate to severe VI and 32.4 million were blind (2). With rising life expectancy and population expansion globally, the prevalence of eyesight loss is expected to climb. According to the World Health Organization, roughly 90% of people with visual impairment live in low-income areas, posing challenges for social and economic development in these places (3).

Early studies have shown that ~10–20% of school-aged children in developing countries face common visual impairment problems (visual acuity [VA] ≤ 6/12 in better eye) (4–6). In most settings, about 90% of these visual impairment among school-aged children are caused by refractive error (7). Visual acuity has been widely used as a proxy measure of refractive error in children in previous studies (6) and to estimate the prevalence of myopia in a large population of people who undergo vision screening when refraction tests are not feasible. In most cases, children's visual impairment can be easily corrected by the timely and proper fitting of quality glasses (8). Unfortunately, studies in a variety of developing countries have shown that 35–85% of individuals with refractive error do not have glasses (9–11).

Many studies have examined the potential negative impacts of children's vision problems (12–14). Common but untreated cases of visual impairment can lead to a variety of broader problems. For example, a study of 3–16 years old children in the UK showed that visual impairment had negative effects on quality of life (15). Other studies showed that the school performance of students were affected when they needed glasses but did not have them (16, 17), and such poor academic performance might limit their future educational attainment, career prospects and lifetime earnings (18).

The prevalence of children with visual impairment appears to be higher in rural China than in other countries (12, 17). A recent study in the same area as our study showed that about 25% of students in grades 4 and 5 had myopia (6). However, a recent investigation in rural China found that fewer than one-third of children needing glasses had glasses and even fewer wore them (19). According to Yi (11), more than 85% of children in rural China with myopia did not wear glasses.

Several studies have reported factors associated with visual impairment and prescription glasses ownership among school-aged children (20, 21). However, most of these studies only examined correlations with gender and age (22, 23), living environment (i.e., living at home vs. boarding at school), and family environment (such as parental education, migration status and family wealth) (6). Some studies investigated behavior factors (such as reading, doing homework and using computer) associated with myopia (24, 25).

Previous studies have not fully addressed the potential impacts of socioeconomic factors on students' vision impairment and glasses ownership, such as county-level income, population density and industrial structure. Earlier studies have demonstrated that the prevalence of visual impairment is closely linked with socioeconomic realities, which has been described as both the cause and consequence of poverty and the lack of accessibility of vision health services (17, 26). However, the relations between the levels of socioeconomic development and the rates of visual impairment and glasses ownership for a particular county remains unclear. Specifically, the gross domestic product (GDP) per capita is commonly used as a macroeconomic indicator. The population density is an indicator of regional resource needs. The industrial structure is a combination of social and economic factors, which can measure the degree of development of the related industries. To our knowledge, previous studies have not examined the relations of these three indicators with the visual impairment and glasses ownership.

As the importance of vision health, the National Health Commission of People's Republic of China announced on 2022 plans“the 14th 5-Year Plan for National Eye Health (2021–2025),” supported by national leader Xi Jinping (27). Actions will sets a target to reduce the national prevalence of visual impairment and to improve the correction rate of visually impaired students. The Chinese government has provided policy and financial support for the prevention and control of students visual impairment. However, it is important that counties of different economic levels set targets that take into account varying disease burdens, diagnostic capacities, and the access to cost-effective interventions. A clearer understanding of the relationships between county-level prevalence of visual impairment, glasses ownership rate and socioeconomic factors could inform resource allocation priorities for the county-level public health planning.

To fill the knowledge gap, the overall goal of this study is to portray disparities in visual health levels and visual corrective behaviors among rural students in counties with different development levels, and investigate the individual characteristics and county-level socioeconomic factors associated with them. Three specific objectives were brought up to meet this goal. First, the study describes the prevalence of visual impairment of rural students in grades 4–9. Second, the study describes the rate of glasses ownership among students with visual impairment. Finally, the study examines the extent to which county-level socioeconomic status and individual characteristics can explain variations in visual impairment and/or glasses ownership rate at the individual level.

## Materials and methods

### Sampling and setting

The data of this study was collected in rural areas of a western Chinese province, Shaanxi. Shaanxi province was a typical impoverished area in northwest China and had a total population of 38.5 million. As in 2015, Shaanxi's GDP per capita was USD 6,965, which was ranked 14th among China's 31 provincial administrative regions according to China National Statistics Yearbook in 2016.

Ten prefectures in Shaanxi Province are divided into three regions: northern, central, and southern. According to the number of prefectures in each region, we randomly selected prefectures from the three regions, including two prefectures in the northern region, one prefecture in the central region, and one prefecture in the southern region. We then obtained a list of counties for these four prefectures and randomly selected counties from each prefecture, resulting in a total of thirteen sample counties. Finally, we included all school students in grades 4–9 in the sample counties as our study sample.

The data analyzed in this study was drawn from the database of Northwestern Rural Children Vision Centers (VC). These VCs were established jointly by the Center for Experimental Economics in Education of Shaanxi Normal University and county-level organizations such as the local Ministry of Education or county hospitals.

Each VC of the county hospital had three staff members, including one ophthalmic specialist and two ophthalmic nurses. All staff members received formal refraction training at Zhongshan Ophthalmic Center in Guangzhou, China. After the training, all qualified employees were certified as national refractive therapists and optometrists by the China Ministry of Labor and Social Security. After the certification, staff members also received 1 month of supervised practical training in their home counties, where each staff member screened and measured hundreds of children from local schools for visual impairment and underwent hands-on instruction in glasses dispensing. Afterwards, the trained staff members carried out the vision screening test for grades 4–9 students in the sample counties.

The vision screening test and survey questionnaires were conducted by VC staffs on a rolling basis. Specifically, the in-school screening was implemented by the vision center staff every week except during summer and winter vacations. The staff carried out the screening 1–2 times per week (covering 2–4 schools) and finished one-round of screening in one town per month. Through this method, it took about 1 year to complete one round of the vision screening for all eligible children in a particular county. The second round and subsequent rounds of vision screening followed the same procedure.

The data used in this study came from the database from 2014 to 2019, covering 99,670 primary and secondary students in 13 counties in Shaanxi Province (Table 1). Among these, more than 50% of students were male, a ratio similar to those found in most poor areas in China (28) due to selective male birth. The sample students were in grades 4 through 9 (Table 1).

**Table 1:** Sample description.

**Sample county**	**Sample province**	**No. of sample schools**	**No. of sample students**	**Grade**	**Boys (%)**	**Survey year**
County 1	Shaanxi	67	9,358	4–9	49.7	2017–2019
County 2	Shaanxi	28	8,210	4–9	51.4	2017–2019
County 3	Shaanxi	31	6,478	4–9	51.1	2018–2019
County 4	Shaanxi	34	11,058	4–9	55.5	2018–2019
County 5	Shaanxi	15	3,609	4–9	52.3	2018
County 6	Shaanxi	42	11,571	4–9	53.8	2017–2018
County 7	Shaanxi	11	5,306	4–9	51.7	2019
County 8	Shaanxi	57	9,728	4–9	53.0	2016–2017
County 9	Shaanxi	38	8,124	4–9	55.0	2018–2019
County 10	Shaanxi	75	16,505	4–9	53.9	2014–2019
County 11	Shaanxi	11	2,894	4–9	60.8	2019
County 12	Shaanxi	21	3,627	4–9	56.0	2015–2016
County 13	Shaanxi	15	3,202	4–9	53.3	2017–2018

### Data collection

This study collected three types of data: school-based vision test results, student-level sociodemographic and county-level socioeconomic information. Children's vision test data and sociodemographic information were collected following a two-part survey protocol in both primary and secondary schools. First, a visual acuity assessment was carried out for all sampled children in school. Second, at the time of visual test, VC staffs administered a simple questionnaire to all sampled children to collect information on their age, gender, and self-reported ownership of glasses.

#### School-based visual acuity assessment

Students also were administered with a visual acuity screening test. The screening test was performed by a team of one optometrist, two nurse. Visual acuity was tested separately for each eye at a distance of 4 m using Early Treatment Diabetic Retinopathy Study (ETDRS) charts in a well-light indoor areas. The chart has 14 rows of optotypes (represented by capital letter E) with 5 optotypes pointing (randomly) in different directions in each row. The sizes of the optotypes become smaller as onemoves fromthe top of the chart to the bottom. Visual acuity (VA) is recorded as 6/X, in which X varies between 60 (at the very top) and 3 (at the very bottom) when tested at a distance of 4 m. Each student started testing fromthe top row (6/60). If the orientation of at least four of the five optotypes was correctly identified the student was re-examined on row 4 (6/30). If one or no optotypes was missed on row 4 the testing resumed at row 7 (6/15) continuing to row 11 (6/6). A failure was defined as an inability to correctly identify the orientation of at least four of the five optotypes in a given row. The row immediately above the failed row was tested until the student identified at least four of the five optotypes in a row. The lowest row read successfully was assigned as the visual acuity for the eye undergoing testing. If the top row was missed at 4 m, the student was advanced to 1 m with progression down the chart as described above and the visual acuity recorded was divided by four. The meaning of the visual acuity fraction 6/X is that the subject can correctly identify at a distance of 6 m what a normal person can see at X meters.

By the convention of the ophthalmology field (29–31), visual impairment is defined as uncorrected visual acuity of ≤ 6/12 of better eye. In analysis, we define visual impairment as a binary variable that takes the value of one if the student has visual impairment and zero if the student does not.

#### Personal sociodemographic information collection

Simple questionnaires were distributed to all sample students. Due to the long duration of each child's vision screening, we only collected key personal sociodemographic information during the vision test, including grade, gender (male = 1), glasses ownership (own glasses = 1), and school type (rural school = 1). In the process of vision screening, the staff member required students who had glasses to wear them. By observing whether a student wore glasses during the vision test, we define glasses ownership as a binary variable that takes a value of one if a student owned a pair of glasses and zero if the student did not.

#### Socio-economic development information collection

The county-level socio-economic development data were collected from China's National Statistical Yearbooks, including county-level per capita GDP, population density, and tertiary industry share. To better describe the results, we transform variables of per capita GDP and population density to a logarithmic scale.

### Statistical methods

To achieve the three objectives of the study, we first use descriptive analysis to examine the prevalence of visual impairment across all sample students, as well as stratified by grades and genders (Objective 1), the grade- and gender-specific rates of glasses ownership among children who needed refractive correction (Objective 2), and the unmet needs for glasses among all sample students. Visual impairment and glasses ownership are both dummy variables.

We then use multiple logistic regression to assess the extent to which the county-level socio-economic development factors and individual characteristics could explain the individual-level variations in visual impairment and glasses ownership (objective 3). In Multiple logistic regression, the covariates were controlled. Variables include school type (rural school = 1), grade (4-9), gender (male = 1), and moderate or severe poor (Yes = 1). The *P*-value of < 0.1 was regarded as a significant difference. All analyses were performed using Stata 15.1 (Stata Corp, College Station, Texas, USA).

## Results

Table 2 shows the prevalence of visual impairment and glasses ownership in different regions. The overall prevalence of visual impairment in the sampled area was 30.4%, with large variation among different counties, ranging from the lowest of 21.0% in county 9 to the highest of 59.5% in county 13. In terms of glasses ownership, the overall rate of glasses ownership of the visual impairment sample was only 39.7%, which also varied greatly among different counties, with the lowest being 12.4% in county 8 and the highest being 57.7% in county 1.

**Table 2:** Distribution of visual impairment and glasses ownership in different regions (*n*/%).

**Sample county**	**No. of sample students**	**Visual impairment** **(*n*, %)**	**Glasses ownership among visual impairment sample (*n*, %)**
County 1	9,358	3,086 (33.0)	1,781 (57.7)
County 2	8,210	2,891 (35.2)	1,140 (39.4)
County 3	6,478	1,899 (29.3)	1,271 (66.9)
County 4	11,058	4,153 (37.6)	1,881 (45.3)
County 5	3,609	1,193 (33.1)	423 (35.5)
County 6	11,571	2,664 (23.0)	839 (31.5)
County 7	5,306	1,563 (29.5)	693 (44.3)
County 8	9,728	2,693 (27.7)	335 (12.4)
County 9	8,124	1,706 (21.0)	585 (34.3)
County 10	16,505	5,370 (32.6)	1,982 (36.9)
County 11	2,894	812 (28.1)	331 (40.8)
County 12	3,627	992 (27.4)	370 (37.3)
County 13	3,202	1,296 (59.5)	409 (31.6)
Total	99,670	30,332(30.4)	12,040 (39.7)

**Figure 1:**
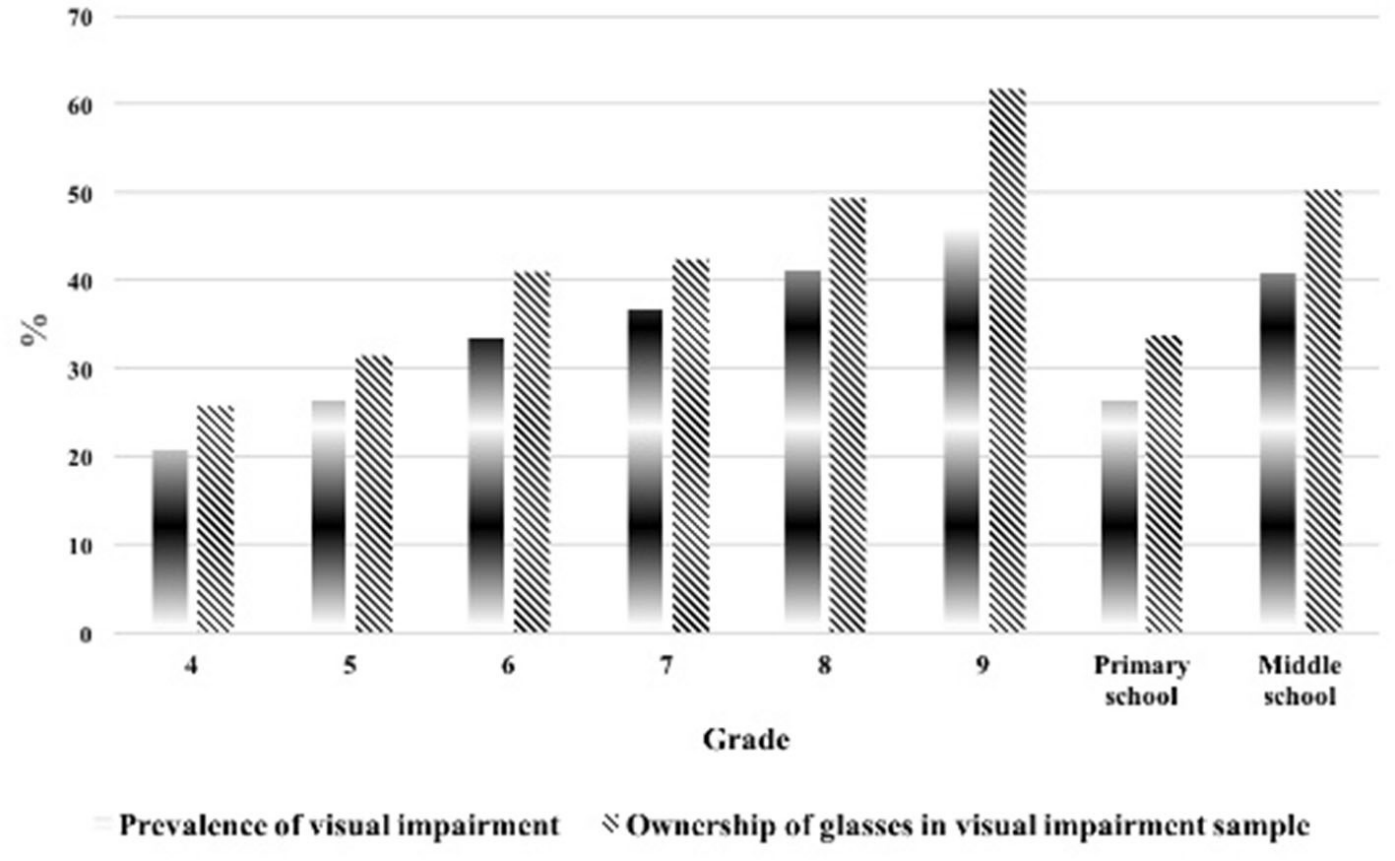
Prevalence of visual impairment of all sample and glasses ownership in visual impairment sample by grades.

Table 3 shows the prevalence of visual impairment and glasses ownership in the visual impairment sample stratified by grade and gender. First, the prevalence of visual impairment differed by gender, and the rate in girls was higher (34.3%) than that in boys (27.0%). We also found that the prevalence of visual impairment increased with grade levels, from 20.6% in the fourth grade to 46.4% in the ninth grade (Figure 1). Second, the rate of glasses ownership in girls (40.6%) was higher than that in boys (38.8%) among students with vision impairment. The rate of glasses ownership also increased gradually with the grade levels, from 25.7% in the fourth grade to 50.2% in the ninth grade (Figure 1).

**Table 3:** Grade-specific prevalence of visual impairment and glasses ownership by gender.

**Grade**	**All sample**	**Visual impairment**			**Ownership of glasses in visual impairment sample**		
		**All** **(*****n*****, %)**	**Boys** **(*****n*****, %)**	**Girls** **(*****n*****, %)**	**All** **(*****n*****, %)**	**Boys** **(*****n*****, %)**	**Girls** **(*****n*****, %)**
4	25,379	5,237 (20.6)	2,493 (18.5)	2,744 (23.0)	1,346 (25.7)	710 (28.5)	636 (23.2)
5	24,603	6,492 (26.4)	3,046 (23.6)	3,446 (29.5)	2,041 (31.4)	973 (31.9)	1,068 (31.0)
6	22,063	7,392 (33.5)	3,461 (29.3)	3,931 (38.3)	3,029 (41.0)	1,393 (40.3)	1,636 (41.6)
7	11,017	4,036 (36.6)	1,940 (32.4)	2,096 (41.7)	1,713 (42.4)	789 (40.6)	924 (44.1)
8	9,979	4,090 (41.0)	1,991 (36.2)	2,099 (46.8)	2,018 (49.3)	877 (44.1)	1,141 (54.4)
9	6,629	3,075 (46.4)	1,436 (40.7)	1,639 (52.8)	1,893 (61.6)	826 (57.5)	1,067 (65.1)
Primary school	72,045	19,121 (26.5)	9,000 (23.6)	10,121 (29.9)	6,416 (33.6)	3,076 (34.2)	3,340 (33.0)
Middle school	27,625	11,201 (40.6)	5,367 (35.8)	5,834 (46.3)	5,624 (50.2)	2,492 (46.4)	3,132 (53.7)
All	99,670	30,332 (30.4)	14,367 (27.0)	15,955 (34.3)	12,040 (39.7)	5,568 (38.8)	6,472 (40.6)

Although the prevalence of visual impairment was highest in middle schools, the unmet need for glasses peaked in primary schools, when more than two-thirds of primary students who needed glasses did not own them. Specifically, this unmet need increased from about 49.8% in middle schools to about 66.4% in primary schools.

Table 4 shows two sets of predictors of visual impairment based on the multivariate model analysis: county-level and individual-level factors. In terms of county-level socio-economic factors, students in counties with high per-capital GDP (OR = 1.47, 95% CI = 1.32–1.62, *p* < 0.001), high population density (OR = 1.52, 95% CI = 1.44–1.61, *p* < 0.001), and high proportion of tertiary industry (OR = 12.14, 95% CI = 7.79–18.91, *p* < 0.001) all had significantly higher risk of visual impairment. In terms of individual characteristics, student attending county schools had higher risk of visual impairment than students attending rural schools (OR = 0.96, 95% CI = 0.92–0.99, *p* = 0.045); students in higher grades were more likely to have visual impairment (OR = 1.32, 95% CI = 1.31–1.33, *p* < 0.001); and girls had higher risk of visual impairment than boys (OR = 0.69, 95% CI = 0.67–0.71, *p* < 0.001).

**Table 4:** Relationship between prevalence of visual impairment and county characteristics.

**Variables**	**Odds ratio**	**95% Confidence interval**	***P*-value**
Per capita GDP	1.47	(1.32–1.62)	< 0.001^***^
Population density	1.52	(1.44–1.61)	< 0.001^***^
Proportion of tertiary industry	12.14	(7.79–18.91)	< 0.001^***^
School type (Rural school = 1)	0.96	(0.92–0.99)	0.045^**^
Grade (4–9)	1.32	(1.31–1.33)	< 0.001^***^
Gender (Male = 1)	0.69	(0.67–0.71)	< 0.001^***^
Screening for rotation fixed effect	YES		
Year fixed effect	YES		
County fixed effect	YES		
Observations	99,670		
*R*-squared	0.05		

^*^P ≤ *0.1;* ^**^P ≤ *0.05;* ^***^P ≤ *0.01*.

Based on the multiple regression model analysis, Table 5 shows the characteristics that were significantly associated with glasses ownership among students with poor vision. In terms of county-level factors, students in counties with high population density (OR = 1.65, 95% CI = 1.50–1.81, *p* < 0.001) and high proportion of tertiary industry (OR = 16.10, 95% CI = 5.66–45.78, *p* < 0.001) had significantly higher rate of glasses ownership. The exception is that the per capita GDP was not associated with the rate of glasses ownership. In terms of individual characteristics, students attending county schools had significantly higher rate of glasses ownership than those in rural schools (OR = 0.62, 95% CI = 0.57–0.67, *p* < 0.001); students attending higher grades were more likely to own glasses (OR = 1.40, 95% CI = 1.37–1.42, *p* < 0.001); girls had higher rate of glasses ownership than boys (OR = 0.94, 95% CI = 0.89–0.99, *p* = 0.025); and students with moderate or severe visual impairment were more likely to own glasses (OR = 5.59, 95% CI = 5.24–5.95, *p* < 0.001).

**Table 5:** Relationship between glasses ownership and county characteristics.

**Variables**	**Odds ratio**	**95% Confidence interval**	***P*-value**
Per capita GDP	1.05	(0.77–1.42)	0.751
Population density	1.65	(1.50–1.81)	< 0.001^***^
Proportion of tertiary industry	16.10	(5.66–45.78)	< 0.001^***^
School type (Rural school = 1)	0.62	(0.57–0.67)	< 0.001^***^
Grade (4–9)	1.40	(1.37–1.42)	< 0.001^***^
Gender (Male = 1)	0.94	(0.89–0.99)	0.025^**^
Moderate or severe poor (Yes = 1)	5.59	(5.24–5.95)	< 0.001^***^
Screening for rotation fixed effect	YES		
Year fixed effect	YES		
County fixed effect	YES		
Observations	30,332		
R-squared	0.21		

^*^P ≤ *0.1;* ^**^P ≤ *0.05;* ^***^P ≤ *0.01*.

## Discussion

This study investigated the association of visual impairment and glasses ownership with county-level socioeconomic status in rural China. Three variables were used to characterize the county-level factors: per-capita GDP, population density and share of tertiary industry. These factors may affect the rate of vision impairment and glasses ownership through their effects on the accessibility and affordability of vision health services for students in rural China.

First, we found that 30.4% of rural students had visual impairment, which is consistent with the results of previous studies (32, 33). Furthermore, the rate of correction was much lower than the prevalence of poor vision. Overall, only 39.7% of visually impaired students owned and wore glasses.

Second, the results showed that the visual impairment of rural students was significantly associated with county-level socioeconomic status, including per capita GDP, population density and the proportion of tertiary industry. This finding is consistent with previous reviews on this topic (34, 35). Possible explanations are that students in wealthier areas may have more access to electronic products and spend more time on TV or computer screens than their poorer counterparts (25, 36), and students in larger population areas may face greater academic competition and burdens, resulting in less time for physical activities (37, 38). These two factors both have been proved to be related to visual impairment (25, 36, 38).

Third, we also found a significant positive correlation between glasses ownership and the population density or the proportion of tertiary industry. This finding suggests that vision care services are more accessible in high-population areas and in more developed areas with higher share of tertiary industries. These are also consistent with those found in the literature (34). Factors of two aspects may contribute to these correlations. On the demand side, the high population density increases the demand for vision care services, thus promoting their development. On the supply side, the quantity and quality of vision care services can be improved by the development of tertiary industry.

Fourth, the results showed that both visual impairment and glasses ownership were significantly associated with higher grade levels. The higher incidence of visual impairment in senior students may be because they had higher academic pressure and spent more time studying in close proximity, which was a risk factor for visual impairment (7, 12). In addition, study pressure not only caused visual impairment, but also contributed to the higher rate of glasses ownership. Senior students may realize that the poor vision is negatively and significantly correlated with academic performance. Therefore, they are more likely to purchase and wear glasses to cope with the heavier academic burden (6, 17).

It's worth noting that, among counties in rural areas, while the prevalence of visual impairment was highest in grade 9, the unmet need for glasses peaked in primary schools, in which 66.4% of students who needed glasses not having them. This result is different from that in urban areas, where the unmet needs for glasses peaked in junior high school (34). The possible explanation is that, comparing to urban parents, rural parents may not be aware of their children's vision problems or mistakenly believe that wearing glasses will deteriorate children's vision when children are too young or only have modest myopia (6, 19).

Consistent with previous studies (34, 39, 40), we found higher rates of visual impairment (34.3%) and glasses ownership (27.0%) among girls than boys. Compared to boys, girls may spend more time studying or working in close proximity and spend less time outdoors (21, 31), which is associated with visual impairment (41, 42), and may lead to a higher demand for glasses. Another reason is that higher rates of visual impairment lead to poorer vision in girls than boys, and worse vision leads to higher rates of glasses ownership in girls.

The study has two strengths. First, the study sample included a population-representative sample of 99,670 students drawn from 445 schools in 13 counties between 2014 and 2019. This sample size is the largest reported in the literature on this issue in rural areas. Second, the dataset included both the individual- and county-level factors, which allowed us to examine not only the correlation of the prevalence of visual impairment and glasses ownership with individual characteristics, but also their relationships with county-level socioeconomic status in rural China, such as county-level income, population density, and industrial structure.

The study has a few limitations. First, the sampling frame of this study was limited to one particular province, which may not be representative of the socially and geographically complex country. Therefore, the research conclusions should be applied with cause to other countries and ethnic groups. Second, we only studied children's glasses ownership behavior, and children's glasses-wearing behavior should also be investigated in future studies. Third, self-reported recall data about the other variables, as adopted by most studies of visual acuity (6, 12, 16), depend on the reliability of informants; this issue may be greater when younger children are involved, as in the present case. Given the resource limitations to researchers following a large cohort of young children, the self-reported recall was determined to be the best method for our study's visual acuity data collection.

Despite these limitations, we believe our study has important implications for social planners. While the incidence of myopia has become more and more serious with economic development, the good news is that the accessibility to correction and treatment for myopia has also increased. However, the relevant policies of vision protection still need to be further improved by the education, medical and public health departments, and the vision health management services for adolescents, especially those in rural areas, should be strengthened. For example, universal vision screening should be integrated into the in-school physical examination plan, and community-based vision healthcare campaigns should be carried out routinely to eliminate common misunderstandings regarding vision care.

Given the low rate of glasses ownership among visually impaired students in rural areas, additional investment is needed to increase the accessibility and affordability of vision care services. On the one hand, as for accessibility, the government, education department and county central hospitals should cooperate to establish vision centers in rural areas. Vision centers are long-term facilities that provide affordable vision care services for local communities. They tend to be promoted to people with uncorrected refractive error and offer a range of services that may include eye examinations, refraction, and glasses dispensing (43).

On the other hand, as for affordability, providing subsidies for the first pair of glasses for rural children will largely reduce the financial barriers to buy the first pair of glasses. Data from another study showed that among children who owned glasses, the median price was ~$60. This is equivalent to nearly half the monthly income of rural families in China. While the cost is a major barrier for rural families in obtaining glasses, high-quality spectacles can be purchased in bulk for < $5, which would make government programs affordable. Therefore, it may be a good solution to include the cost of first pair of glasses for rural students in the reimbursement coverage of China's rural health insurance system.

Finally, in order to reduce disparities in vision health and healthcare service utilization caused by regional differences in socioeconomic development, government healthcare policies or programs should take into account the regional differences in per capita income, population density, and industrial development, and focus on directing public health service resources to poor regions and disadvantaged groups, so as to narrow the gap in vision healthcare services.

## Conclusion

This study contributes to the literature by systematically exploring and confirming the positive association of the visual impairment and glasses ownership with the county-level socioeconomic status in a low-income area through large-scale sampling. The results suggest that the accessibility and affordability of vision care services can be increased by the development of tertiary industry in countries with large populations. Furthermore, our findings provide a theoretical basis for public health policymakers to effectively tailor policies to address vision health issues, which also suggest that increasing the supply of vision care services could theoretically improve myopia correction and treatment in rural areas.

Based on the results of this study and the characteristics of vision correction behaviors, we recommend that health and education policymakers in China take steps to integrate vision care into the public health and education agenda, especially in rural schools. Specifically, universal vision screening should be included in the current in-school physical examination program that requires one school year to ensure that rural schools in China can implement regularly and high-quality vision screening for students.

Second, the results show that the provision of vision health services is an important reason for limiting the glasses ownership of visual impairment students compared to income. Therefore, on the supply side in the future, relevant policy makers should consider increasing access to high-quality, low-cost or free glasses for rural school children, especially in the least densely populated rural areas.

## Data availability statement

The raw data supporting the conclusions of this article will be made available by the authors, without undue reservation.

## Ethics statement

This study was provided by Stanford University Institutional Review Board (Registration number: ISRCTN03252665, registration site: http://isrctn.org) and Sun Yat-sen University (Registration number: 2013MEKY018). Written informed consent to participate in this study was provided by the participants' legal guardian/next of kin.

## Author contributions

HG and YZ conceived the study and drafted the manuscript. HG, YZ, ZW, YD, and JX designed the questionnaires. ZW, XC, and JX performed the analysis and interpreted the results. All authors critically revised the manuscript and approved the final manuscript as submitted.
